# Effectivity comparison between three different enamel remineralizing agent postfix orthodontic treatment

**DOI:** 10.4317/jced.55933

**Published:** 2019-10-01

**Authors:** Ari Triwardhani, Irwadi Djaharu’ddin, Putu-Andy Herawan

**Affiliations:** 1 Orthodontic Department, Faculty of Dental Medicine, Campus A Universitas Airlangga Jl. Indonesia

## Abstract

**Background:**

This study aims to compare the effectiveness of topical fluoride application, a toothpaste containing synthetic hydroxyapatite with one containing calcium sodium phosphosilicate (CSPS) in remineralizing enamel after the debonding process.

**Material and Methods:**

The study constituted experimental laboratory research incorporating posttest-only control group design. A metal bracket was placed on the buccal surface of 40 premolar teeth and immersed in artificial saliva. After 1 month, the bracket was debonded. The teeth were then randomly divided into four groups, namely (n = 6) the control group (C), the 1st treatment group (T1), the 2nd treatment group (T2), and the 3rd treatment group (T3). There was no treatment of C group. For the T1 group, topical application of fluoride was conducted. For the T2 group, toothpaste containing synthetic hydroxyapatite was applied by brushing the teeth twice a day. The same treatment was administered to the T3 group, but using a different toothpaste containing CSPS. After a treatment of 14 days, the T1, T2, and T3 groups were prepared before being observed with a scanning electron microscope (SEM). The SEM data were examined visually and scored using an enamel surface index (ESI), an enamel damage index (EDI), and an enamel remineralization index. The data obtained were analyzed by means of Kruskal–Wallis and Mann–Whitney tests. The statistical significance value was *P*< 0.05.

**Results:**

A review of the ESI and IRE scores showed that, compared to the C group, all treatment groups recorded a significantly lower score, with T2 registering the lowest. With regard to the EDI scores, only the T1 group showed no significant difference to the C group.

**Conclusions:**

Sensitive toothpaste containing synthetic hydroxyapatite and CSSP proved to be more effective in forming the remineralization layer on tooth surfaces compared to topical fluoride application.

** Key words:**Calcium sodium phosphosilicate, debonding process, enamel remineralization, synthetic hydroxyapatite, topical fluoride application.

## Introduction

Fix orthodontic treatment currently represents the preferred option for both orthodontists and patients. Compared to removable orthodontic appliances, the fix varieties have several advantages including providing better results and producing more complex tooth movement, while not requiring full patient compliance as is the case with removable orthodontic treatment (1,2).

However, the use of adhesive material to attach the bracket to the tooth surface in fix orthodontic treatment creates a problem on termination as it can cause enamel destruction during the debonding process and the removal of residual adhesive (3). Enamel tissue destruction can occur if the shear bond strength of the adhesive material was greater than that of the enamel, some of which would be lost during bracket removal.

To anticipate the possibility of enamel destruction during debonding, the application of topical fluoride administration immediately after cleaning of the enamel surface was recommended (4). The use of fluoride varnish, chlorhexidine, xylitol, or remineralization products containing calcium can also help to accelerate enamel remineralization.

Several products containing new active compounds in addition to fluoride have been developed promising more rapid tooth remineralization. In recent years, the number of manufacturers introducing sensitive toothpaste products which promote enamel remineralization in the market has increased. Some of these products contain synthetic hydroxyapatite and calcium sodium phosphosilicate (CSPS).

The use of synthetic hydroxyapatite in sensitive toothpaste products has recently increased. According to Jeong *et al.* (5) synthetic hydroxyapatite in toothpaste can act as an abrasive material to remove discoloration of tooth surfaces and stimulate remineralization in incipient caries.

CSPS was 1st introduced to address the issue of sensitive teeth, but recently its ability to stimulate tooth remineralization has been reported (6). CSPS offered a more rapid remineralization than fluoride by closing the open dentine tubules and forming hydroxycarbonate apatite crystals immediately on exposure to body fluids and saliva (7,8).

The purpose of this study was, therefore, to compare the effectiveness of topical fluoride application with high doses of sodium fluoride, a sensitive toothpaste containing synthetic hydroxyapatite and one containing CSPS in remineralizing tooth enamel after the debonding process in postfix orthodontic treatment.

## Material and Methods

**Figure 1:**
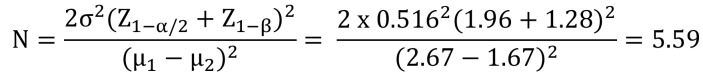
Formula.

This was an experimental laboratory study incorporating posttest-only control group design. The sample used consisted of 40 extracted premolar teeth meeting the inclusion criteria of being in good condition with a caries-free enamel buccal surface. A Mini Master series slot 0.022 (American Orthodontics, Washington, USA) metal bracket was placed on the buccal surface of the teeth using O phosphoric acid as an etching agent (Dental Life Science, Wigan, UK) and Xeno Ortho adhesive paste (Denstply, Tokyo, Japan), before being immersed in artificial saliva for 1 month at 37°C. During this period of immersion, the artificial saliva was replaced once every 5 days. After a month, the brackets were debonded, and the remaining adhesive was removed with pliers (Hu- Friedy, Chicago, USA). The teeth were subsequently randomly divided into four groups (n = 6): A control group (C), a 1st treatment group (T1), a 2nd treatment group (T2), and a 3rd treatment group (T3). The sample size is calculated using the following formula (9): (Fig. 1):

No treatment was applied to the control group. After debonding, the teeth were prepared using a disk diamond bur (Denstply, Tokyo, Japan) with a straight angle low-speed handpiece (NSK, Tokyo, Japan) before being observed through a Carl Zeiss EVO MA 10 Scanning Electron Microscope (SEM) (ZEISS, Oberkochen, Germany) at ×500 magnification. For the T1 group, topical application of fluoride Clinpro (3M ESPE, California, USA) was conducted for 15 s following debonding. After application, the teeth were immersed in artificial saliva for 14 days. Postdebonding treatment of T2 group consisted of the application of toothpaste containing synthetic hydroxyapatite (Unilever, Jakarta, Indonesia) by brushing the teeth twice a day with a minimum time span of 7 h between treatments for 14 days. The same treatment was performed on the T3 group, but using a different toothpaste containing CSPS (GlaxoSmithKline, Jakarta, Indonesia). During the treatment of both the T2 and T3 groups, the teeth were immersed in artificial saliva. After 14 days of treatment, the T1, T2, and T3 groups were also prepared using a disk diamond with a straight angle low-speed handpiece, before being observed with an SEM.

The SEM data were visually examined by two experts without any grouping and interventional information data, and then scored using three different scoring systems: The enamel surface index (ESI), the enamel damage index (EDI), and the enamel remineralization index (ERI), which constituted a modified ESI with several points adjusted for the purposes of this study.

ESI was 1st constructed by Zachrisson and Arthun (10) and later used by Pont *et al.* (11) and Sessa *et al.* (12):

0. Perfect surface with no scratches and distinct intact perikymata

1. Satisfactory surface with fine scratches and some perikymata

2. Acceptable surface with several marked and some deeper scratches, no perikymata

3. Imperfect surface with several distinct deep and coarse scratches, no perikymata

4. Unacceptable surface with coarse scratches and deeply marked appearance.

EDI was first propounded by Schuler and van Waes (13) and later adopted by Alessandri Bonetti *et al.* (14) and Baumann *et al.* (15):

Acceptable surface with fine scattered scratches

Rough surface with numerous coarse scratches or minor grooves

Surface with coarse scratches with wide grooves and enamel damage visible to the naked eye.

ERI was made by modifying the ESI as both ESI and EDI remained inadequate at observing the remineralization process by means of SEM examination in this study. This scoring system consists of five scales:

0. Surface with coarse scratches and enamel damage visible to the naked eye

1. Surface with several coarse scratches, but subject to finer enamel damage

2. Surface with much finer scratches and enamel damage, remineralization covering part of its area

3. Surface with remineralization covering almost the entire tooth surface without distinct enamel damage, but visibly rough

4. Surface with remineralization covering the entire tooth surface with smoother surface visible

5. Remineralization layer forming a smooth surface covering the entire tooth.

-Statistical analysis

The data obtained were analyzed with SPSS version 21 (IBM, New York, USA) using Kruskal–Wallis and Mann–Whitney tests. The statistical significance value was *P* < 0.05.

## Results

-Visual examination

**Figure 2:**
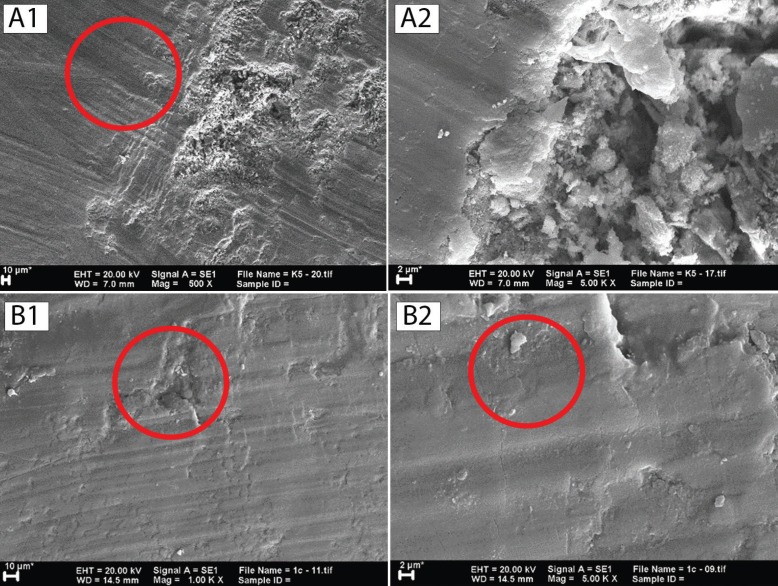
(A) The appearance of a sample of the C group. (A1) At ×500 magnification, rough scratches (red circles) with deep and rough erosion were observed in the center of the sample. (A2) At magnification of ×5000, deep and rough erosion was clearly visible. (B) The appearance of a sample of the T1 group. (B1) At ×1000 magnification, erosion was still evident (red circles) although smaller and shallower than C group. (B2) At ×5000 magnification, smoother scratches were visible (red circle).

**Figure 3:**
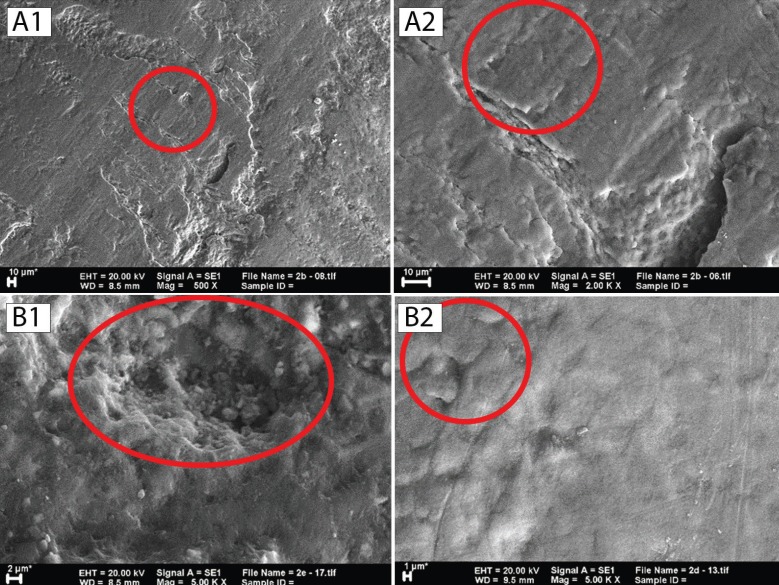
(A) The appearance of a sample of the T2 group. (A1) At ×500 magnification, fine lines were visible (red circle). (A2) At ×2000 magnification, a clearer picture was produced which indicates the lines were formed of remineralizing material deposit (red circle). (B) The appearance of a sample of the T2 group. (B1) At ×500 magnification, a shallow erosion with smooth rounded edges was visible (red circle). (B2) ×5000 magnification revealed scales (red circle).

**Figure 4:**
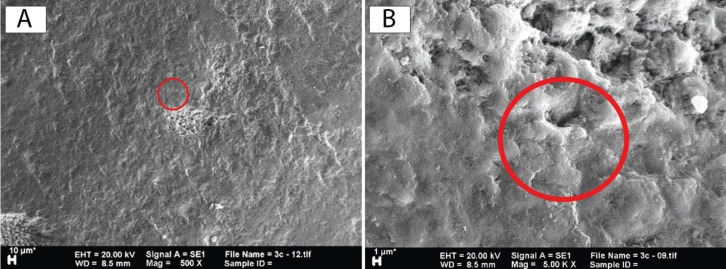
The appearance of a sample of the T3 group. (A) At ×500 magnification, a remineralization layer was found to cover almost the entire tooth surface, although some erosion still could be detected (red circle). (B) Magnification of ×5000 could provide a clearer picture of scales (red circle) which caused the remineralization layer to appear rough.

SEM was used to observe differences in the sample surfaces after treatment of the respective groups. Magnification at ×500, ×1000, ×2000, and ×5000 was used in this study. Observation of the C group revealed the appearance of coarse scratches and the extensive presence of deep, rough, and close distance erosion in the around the bracket (Fig. 2A). Observation of the T1 groups showed coarse scratches similar to those found in the C group, although they have a smoother and shallower appearance (Fig. 2B). Observation of the T2 groups revealed different features compared to those of the C and T1 groups as no coarse, deep scratches were detected but, rather, fine line, and shallow-rounded edge erosion which could only be observed at higher magnification (Fig. 3A). A faint, fine scale appearance about 3–5 μm in size was also common to all samples (Fig. 3B), while shallow erosion in the T3 group similar to that found in the T2 group, albeit with smoother round edges, was observed. A stack of scales found in all T3 group samples was larger, about 4–9 μm, and clearer compared to those of the T2 group which gave a rougher impression (Fig. 4).

The data recorded following each score examination ( Table 1) were analyzed using a nonparametric Mann–Whitney test, which can be seen in  Table 2. The ESI and IRE scoring results show that compared to the C group, all treatment groups showed a significantly lower score, with the T2 group registering the lowest score, although no significant difference was found compared to the T3 group. For the EDI scoring result, only the T1 group demonstrated no significant difference to the C group, while the T2 group showed no significant difference compared to its T1 and T3 counterparts.

**Table 1:**
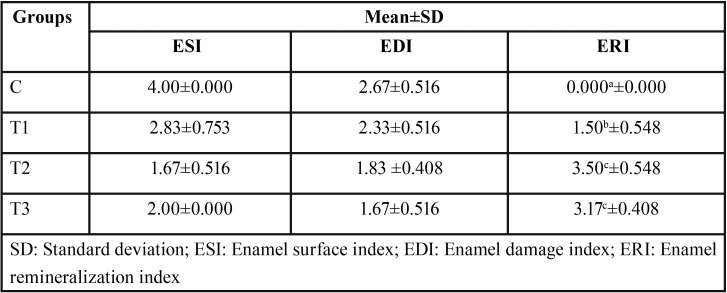
The mean and standard deviation of the control and treatment group enamel surface index, enamel damage index, and enamel remineralization index scoring results.

**Table 2:**
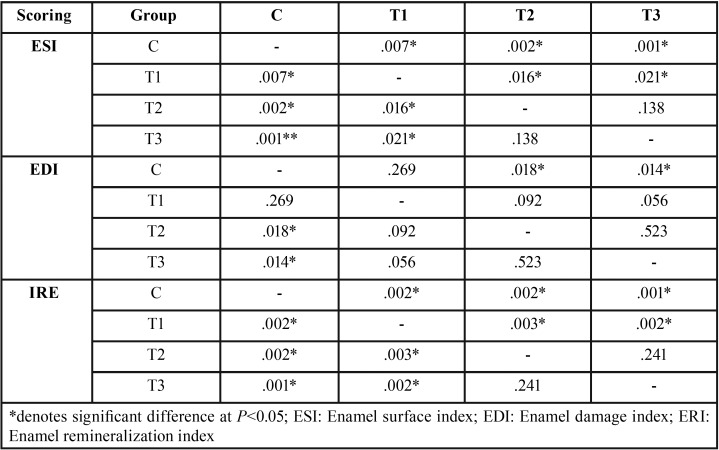
The Mann–Whitney test result of three different scoring system.

## Discussion

Fixed orthodontic appliances have several advantages compared to their removable counterparts. However, they also suffer from their own weaknesses. A good fix appliance requires strong bonding between the tooth surface and the brackets to promote effective tooth movement (3). To achieve such strong bonding, adhesive material was used. In the development of orthodontic treatment, a traditional adhesive system of composite constitutes the primary choice due to its convenience application and attractive aesthetics. However, the use of etching could produce microporosity leading to demineralized enamel white spots which results in a severe problem (16).

Micropores formed on the enamel surface serve as strong attachment points for a composite material which can fill the micropores and bond strongly with enamel when the composite inside has set. This strong bond can cause enamel loss after the debonding process resulting in deep, rough erosion on the enamel surface which will increase the risk of caries (16).

One of the methods used to repair enamel damage is fluoride topical application to the surface of the enamel (4,17). However, this application must be conducted over a prolonged period to produce sufficient remineralization of the damaged enamel surface (17).

Technological development in dentistry has spawned the successful invention of several new materials serving as remineralizing agents, including synthetic hydroxyapatite mineral and CSPS. At present, both materials have been widely used in various sensitive toothpastes due to their ability to close open dentin tubules (18,19).

The purpose of this study is to compare the effectiveness of three remineralization agents in remineralizing enamel after fix orthodontic treatment. The agents studied were high-dose fluoride topical application and two sensitive toothpaste each of which contained synthetic hydroxyapatite and CSPS.

The resulting SEM observation of the C group revealed the presence of enamel damage after the debonding and the adhesive removal processes. This was possibly due to the use of adhesive removing pliers during the removal process causing enamel cracks and damaging the enamel surface.

The observation of the T1 group revealed predominantly superficial milder scratches with smaller and shallower erosion compared to the C group. This demonstrated that a remineralized layer had been successfully formed across the enamel surface, although still insufficient in density to cover all debonding process-induced scratches and erosion. This result reflected the findings of the study by Zabokova-Bilbilova *et al.* (20) which stated that the use of fluoride as a topical application agent requires considerable time to produce ideal remineralization, while the presence of deep, coarse scratches as found in the C and T1 groups was absent from the T2 group. Instead, faint, fine lines, and scales accompanied by shallow rounded-edge erosion was present, possibly due to the uneven accumulation of remineralized tissue on the surface of the enamel.

This conclusion found support in a study by Swarup and Rao (21) which produced a similar result in remineralizing the demineralized premolar tooth by submergence in synthetic nanohydroxyapatite powder and 2% neutral sodium fluoride for 10 days. The results revealed the nucleation of apatitic crystal pores and the pore covering by a dense apatitic layer in the synthetic hydroxyapatite group, while the sodium fluoride group exhibited the presence of uncovered porous surfaces. In addition, there was an increase in the Ca/PO4 ratio and the surface microhardness in the synthetic hydroxyapatite group compared to the sodium fluoride group. Another study conducted by Jeong *et al.* (5) demonstrated that toothpaste containing synthetic nanohydroxyapatite demonstrates strong potential for remineralization, while the addition of fluoride to toothpaste produced no synergic effect on remineralization.

The observation of the T3 group exhibited no deep, coarse scratches such as were found in the C group, although similar shallower erosion was found to be present in the T2 group. Moreover, a larger and clearer appearance of stacked scales, compared to T2 group, was also detected. These were the same uneven, accumulated, remineralized tissue layers found in the T2 group.

This result matched that of the study conducted by Mony *et al.* (22) comparing the remineralizing capacity of two different toothpaste containing NovaMin (CSPS) and fluoride for 15 and 30 days. Toothpaste containing CSPS demonstrates superior remineralization with a significant increase in surface hardness and Ca/PO4 ratio compared to the fluoride group. Other studies by Golpayegani *et al.* (23) which also compared the remineralizing capability of two different toothpaste containing NovaMin and 1.1% neutral sodium fluoride respectively, produced the same result. NovaMin has a better remineralizing effect marked by an increase in surface microhardness. This study also detected no presence of a hypoplastic dots in children’s teeth such as is usually found in the use of conventional toothpaste.

Three scoring examinations used to analyze the SEM result were ESI, EDI, and ERI. The results of ESI and ERI scoring showed a significant difference between the T1 group and both the T2 and T3 groups, but no difference between the T2 and T3 groups. A contrasting result was found in the EDI scoring, which revealed no significant difference between the T1, T2, and T3 groups, but a significant difference between the C group and both the T2 and T3 groups. This was possibly due to the small range of EDI compared to two other scoring systems. The extent of the assessment scope between each scoring range of the EDI system allegedly affected its accuracy when analyzing the SEM result. Therefore, the EDI scoring system was unsuitable for use as a remineralization scoring system in this study.

The ESI and ERI scoring results demonstrated that the material contained in sensitive toothpaste used in both the T2 and T3 groups was more effective at remineralizing compared to the topical fluoride used in the T1 group. This result was supported by the findings of Li *et al.* (24) whose study confirmed that the use of toothpaste containing synthetic hydroxyapatite produced a better remineralization result due to the nanosize of the synthetic hydroxyapatite crystals. Nanohydroxyapatite can mimic the size of natural enamel apatite which allows it to be effectively absorbed by natural human tooth tissue. Other studies also posited that synthetic nanohydroxyapatite also serves as a better source of calcium ion compared to micro-hydroxyapatite which conceded it in sufficing the availability of mineral content needed and delayed the demineralization process, while increasing the remineralization rate (24).

CSPS contained in the toothpaste used in the T3 groups consisted of a bioactive glass capable of forming a carbonated hydroxyapatite (HCA) surface layer with 100–150 µ in thickness in 12–24 h (24). CSPS, known as NovaMin, was reported to be capable of replacing the mineral content of white-spot lesions such as HCA, due to its similarity to enamel crystal structure, and reproducing the lost enamel layer. The most important characteristic of calcium phosphate or calcium fluoride materials is their solubility, despite other calcium being insoluble (22). In a clinical test, toothpaste containing CSPS bioactive glass produced better results in decreasing tooth sensitivity compared to those containing strontium chloride. This material also possessed antimicrobial properties which eliminated 99.99% of the oral pathogens causing periodontal disease and caries (25).

This study concluded that sensitive toothpaste containing synthetic hydroxyapatite and CSPS proved more effective in forming the remineralization layer on the tooth surface compared to high-dose topical fluoride application. The use of sensitive toothpaste containing synthetic hydroxyapatite can form a smoother remineralization layer compared to those containing CSPS, although no significant difference was found.
